# Generation and Application of Monoclonal Antibodies against Porcine S100A8, S100A9, and S100A12 Proteins Using Hybridoma Technology

**DOI:** 10.3390/ijms25021029

**Published:** 2024-01-14

**Authors:** Pengpeng Xia, Xin Ma, Li Yan, Siqi Lian, Xiangyu Li, Yi Luo, Ziyue Chen, Xingduo Ji

**Affiliations:** 1 College of Veterinary Medicine (Institute of Comparative Medicine), Yangzhou University, Yangzhou 225009, China; nn20000413@163.com (X.M.); yanli3559@163.com (L.Y.); mx120180715@yzu.edu.cn (S.L.); lxy2419216465@163.com (X.L.); luoyi_0910@163.com (Y.L.); chenziyue1122@yeah.net (Z.C.); l15852878389@163.com (X.J.); 2 Jiangsu Co-Innovation Center for Prevention and Control of Important Animal Infectious Diseases and Zoonoses, Yangzhou 225009, China; 3 Joint International Research Laboratory of Prevention and Control of Important Animal Infectious Diseases and Zoonotic Diseases of China, Yangzhou University, Yangzhou 225009, China

**Keywords:** S100 proteins, monoclonal antibody, swine health, hybridoma technology

## Abstract

S100A8, S100A9, and S100A12 proteins are important members of the S100 protein family, act primarily as congenital immunomodulators, and are closely related to the occurrence of infectious diseases. There have been few reports on the functional properties of S100A8, S100A9, and S100A12 proteins in swine, but it is certain that porcine S100A8, S100A9, and S100A12 proteins are highly expressed in diseased swine. To address the current lack of reliable and timely detection tools for these three proteins, we generated monoclonal antibodies specific to the porcine S100A8, S100A9, and S100A12 proteins using hybridoma technology. The results of serum sample testing showed that the above monoclonal antibodies specifically recognize the proteins S100A8, S100A9, and S100A12 in the serum and were able to evaluate the content change of these proteins during the infection process. This provides the basis for the use of porcine S100A8, S100A9, and S100A12 in the surveillance and diagnosis of swine diseases and laid a foundation for further understanding their roles in infection, immunity, and inflammation, as well as their potential applications in preventing or treating gastrointestinal tract or inflammatory diseases in swine.

## 1. Introduction

S100A8, S100A9, and S100A12 are EF-hand calcium-binding proteins belonging to the vertebrate-specific S100 protein family. They are mostly expressed in the immune cells and exhibit powerful biological functions by defending pathogens or helping the body fight against diseases [1,2]. Due to the presence of transition metal binding sites, these proteins can chelate metal nutrients necessary for the growth of bacteria through nutritional immunity, including zinc ion (Zn^2+^), manganese ion (Mn^2+^), and copper ion (Cu^2+^), etc., inhibit the growth of pathogenic bacteria and participate in the antimicrobial process [3,4]. The ability to bind to foreign antigens also helps the aforementioned proteins promote superoxide production and antigen clearance by macrophages [3,5]. In addition to binding and invading pathogens, S100A8, S100A9, and S100A12 proteins act as damage-associated molecular patterns (DAMPs) to initiate nuclear factor-kappa B (NF-κB) and mitogen-activated protein kinase (MAPK) signal transduction, inducing the expression of pro-inflammatory cytokines, such as Interleukin 1β (IL-1β), IL-6, and IL-8. They also participate in the regulation of immunity and inflammation via their interaction with the cell surface receptors, such as the receptor for advanced glycation end products (RAGE) and Toll-like receptor 4 (TLR4) [2,4,6].

It is certain that the content of S100A8, S100A9, and S100A12 proteins in serum or fecal are closely related to diseases and can be used as biomarkers for their detection and diagnosis, including rheumatoid arthritis (RA), inflammatory bowel disease (IBD), cancer, etc. [7,8,9]. However, since the biological activities of S100A8, S100A9, and S100A12 proteins are affected by redox conditions, pH, metabolite, and nutrient distribution at the site of infection [10,11], it is unclear whether the changes in protein expression are consistent with their biological activities yet. Fecal calprotectin (S100A8/S100A9 heterodimer) is used as a highly sensitive and non-invasive marker for tracking intestinal inflammation and distinguishing between IBD and irritable bowel syndrome (IBS), while fecal S100A12 is granulocyte-specific and thus more reliable for the clinical detection of bacterial gastroenteritis compared to calprotectin [12,13].

Apart from playing a vital role in human health and diseases, S100A8, S100A9, and S100A12 proteins are closely related to the infection (including bacteria, viruses, and parasites) process in pigs. *Haemophilus parasuis* infection caused an increased mRNA expression of S100A8, S100A9, and S100A12 in the blood and spleen of diseased pigs [14,15]. The transcription activation of S100A12 was positively regulated by C/EBPβ (CCAAT/enhancer-binding protein beta) and activator protein-1 (AP-1) and further participated in the regulation of immune response in pigs. The same regulatory effect may occur in S100A8 and S100A9 to a greater extent because they share a similar expression pattern to the S100A12 gene and have C/EBPβ and AP-1 binding sites in their promoter regions [14,16]. The expression of S100A8, S100A9, and S100A12 are also dramatically induced in porcine whole-blood cultures by both lipopolysaccharide (LPS) and polyinosinic-polycytidylic acid (poly (I: C)) and with porcine circovirus type 2 (PCV-2) infection in vivo [14,15]. So far, however, little has been reported on the functional properties of the proteins S100A8, S100A9, and S100A12 in swine.

In 1975, hybridoma technology was first described by Georges Köhler and Cesar Milstein, and depending on its relatively simple procedure, low cost, and sustainable production, it became one of the most common methods to produce monoclonal antibodies (mAbs) [17]. The wide application of mAbs is crucial for the diagnosis and treatment of diseases, such as cancers and infectious/immune-mediated diseases, and it is also beneficial for the development of vaccines, targeted therapies, and research [18,19,20]. To address the current lack of reliable and timely detection tools for porcine S100A8, S100A9, and S100A12 proteins during the infection process, we generated mAbs specific to these three proteins in this study using hybridoma technology. The results of serum sample testing showed that these mAbs, as described above, specifically identified the proteins S100A8, S100A9 and S100A12 in the serum and were able to evaluate changes in the protein content of S100A8, S100A9, and S100A12 during infection.

## 2. Results

### 2.1. Expression and Purification of the Recombinant S100A8, S100A9, and S100A12 Proteins

**Figure 1:**
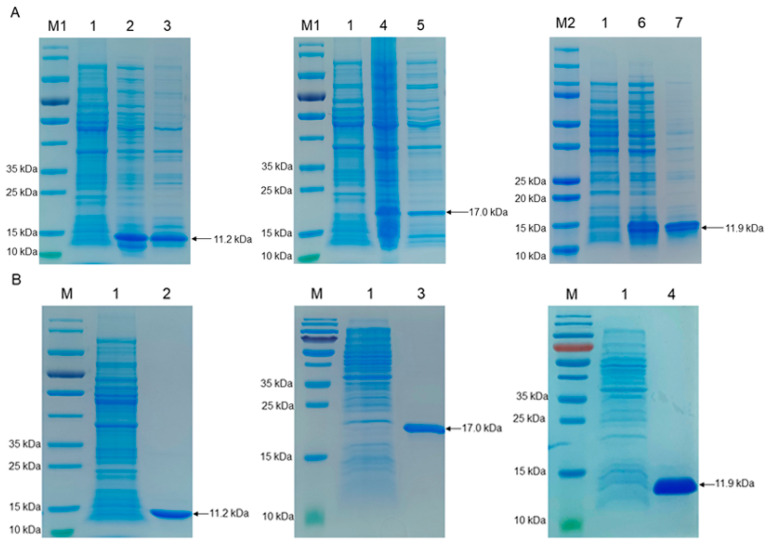
Expression and purification of S100A8, S100A9, and S100A12 proteins were measured by using SDS-PAGE (Coomassie-blue stained). (**A**) Prokaryotic expression of recombinant proteins. M1: protein molecular weight marker (cat#26616, Thermo Fisher Scientific, Waltham, MA, USA); M2: protein molecular weight marker (cat#1610374, Bio-Rad, Hercules, CA, USA); 1: pET28a(+)-BL21(DE3) bacteria lysate; 2: supernatant of pET28a(+)-S100A8-BL21(DE3) lysate; 3: lysate precipitation of pET28a(+)-S100A8-BL21(DE3) bacteria; 4: supernatant of pET28a(+)-S100A9-BL21(DE3) lysate; 5: lysate precipitation of pET28a(+)-S100A9-BL21(DE3) bacteria; 6: supernatant of pET28a(+)-S100A12-BL21(DE3) lysate; 7: lysate precipitation of pET28a(+)-S100A12-BL21(DE3) bacteria; (**B**) Purification of recombinant proteins. M: protein molecular weight marker (cat#26616); 1: pET28a(+)-BL21(DE3) bacteria lysate; 2: the purified S100A8 protein; 3: the purified S100A9 protein; 4: the purified S100A12 protein.

The S100A8, S100A9, and S100A12 proteins are induced with 0.3 mM isopropyl β-d-1-thiogalactopyranoside (IPTG) at 37 °C for 4 h using the strains pET28a(+)-S100A8-BL21(DE3), pET28a(+)-S100A9-BL21(DE3), and pET28a(+)-S100A12-BL21(DE3) (Figure 1A). After cell lysis, the target proteins were purified using a nickel column. The SDS-PAGE results indicated that S100A8 (11.2 kDa), S100A9 (17.0 kDa), and S100A12 (11.9 kDa) proteins appeared with expected sizes and banding patterns (Figure 1B). Notably, the protein mainly exists in the supernatant of cell lysis (Figure 1A) and has a certain bacteriostatic effect on enterotoxigenic *Escherichia coli* (ETEC) F4ac (Figure S1), which proved to be biologically active and optimal for immunizing mice.

### 2.2. Preparation and Production of mAbs

**Figure 2:**
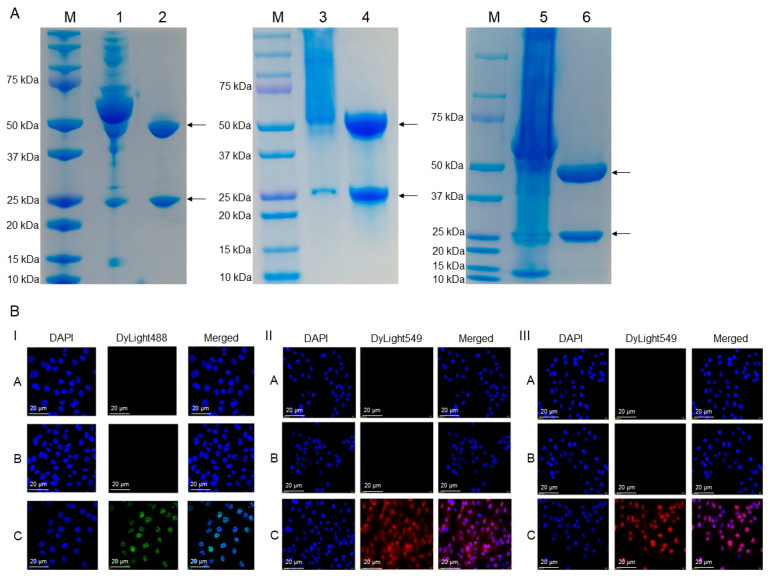
Identification of mAbs against porcine S100A8, S100A9, and S100A12 proteins: (**A**) SDS-PAGE analysis (Coomassie-blue stained) of purified mAbs against porcine S100A8, S100A9, and S100A12 proteins. M: protein molecular weight marker (cat#1610374, Bio-Rad); 1, 3, 5: unpurified mAbs against S100A8, S100A9, and S100A12 proteins; 2, 4, 6: purified mAbs against S100A8, S100A9, and S100A12 proteins using protein A agarose, the lane of the heavy and light chains was annotated with arrows. (**B**) IFA detection of S100A8-F8F9, S100A9-C92H, and S100A12-F5C6 mAbs using pcDNA3.1(+)-S100A8-IPEC-J2, pcDNA3.1(+)-S100A9-IPEC-J2 and pcDNA3.1(+)-S100A12-IPEC-J2 cells, respectively. I. A: PBS; B: Unimmunized mouse serum; C: S100A8-F8F9 monoclonal antibody; II. A: PBS; B: Unimmunized mouse serum; C: S100A9-C92H monoclonal antibody; III. A: PBS; B: Unimmunized mouse serum; C: S100A12-F5C6 monoclonal antibody. DAPI (4′,6-diamidino-2-phenylindole) was used as a nuclear counterstain in fluorescence microscopy. The DyLight 488-Goat Anti-Mouse IgG with green fluorescence was used for S100A8 detection, and the Dylight 549-Goat Anti-Mouse IgG with red fluorescence was used for S100A9 and S100A12 detection.

BALB/c mice immunized with purified porcine S100A8 protein three times at two-week intervals exhibited a higher antibody titer against S100A8 protein in the serum. After four continuous positive selection and freeze–thaw cycles, seven hybridoma cell lines that stably secrete mAbs against porcine S100A8 protein were obtained. Likewise, six hybridoma cell lines for porcine S100A9 protein and four hybridoma cell lines for porcine S100A12 protein survived the rigorous selection. S100A8-F8F9, S100A9-C92H, and S100A12-F5C6 mAbs were selected randomly as the representative samples. The purified mAbs possessing heavy and light chains (50 kDa and 25 kDa, respectively) from the harvested mouse ascites were determined through the use of SDS-PAGE, and the results of the indirect immunofluorescence assays (IFA) show that they can recognize the S100A8, S100A9, and S100A12 protein expressed in pcDNA3.1(+)-S100A8-IPEC-J2, pcDNA3.1(+)-S100A9-IPEC-J2 and pcDNA3.1(+)-S100A12-IPEC-J2 cells, respectively (Figure 2).

### 2.3. Characterization of mAbs against Porcine S100A8, S100A9, and S100A12 Proteins

**Figure 3:**
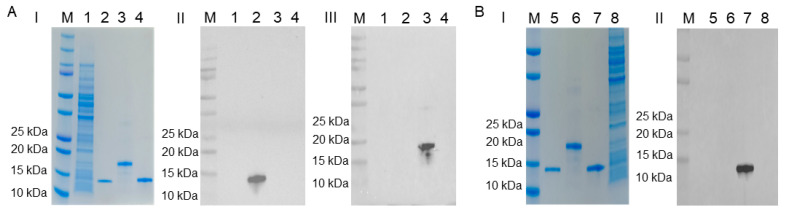
Western-Blot analysis of mAbs against porcine S100A8, S100A9, and S100A12 proteins. M: protein molecular weight marker (cat#1610374, Bio-Rad); (**A-I**, **B-I**) SDS-PAGE analysis of proteins stained with Coomassie blue. (**A-II**) The blot was incubated with S100A8-F8F9 monoclonal antibody; (**A-III**) the blot was incubated with S100A9-C92H monoclonal antibody. 1, pET28a(+)-BL21(DE3) bacteria lysate; 2: Purified S100A8 protein; 3: Purified S100A9 protein; 4: Purified S100A12 protein. (**B-II**) The blot was incubated with S100A12-F5C6 monoclonal antibody. 5: Purified S100A8 protein; 6: Purified S100A9 protein; 7: Purified S100A12 protein; 8, pET28a(+)-BL21(DE3) bacteria lysate.

The Western blot assay results demonstrate that the S100A8-F8F9, S100A9-C92H, and S100A12-F5C6 mAbs can specifically recognize their target proteins (Figure 3). The S100A8-F8F9 monoclonal antibody does not cross-react with the S100A9 and S100A12 proteins of the S100 protein family, nor does the other two mAbs cross-react with the non-target proteins. The binding affinities of S100A8-F8F9, S100A9-C92H, and S100A12-F5C6 mAbs were determined, as shown in Figure S2, and the EC50 values were 0.0629, 0.0412, and 0.4954. The titers of these mAbs in one-day cell culture supernatants and ascites are shown in Table 1. The result of mouse mAbs isotyping revealed that mAbs derived from clones S100A8-C6D2, -C6F6, -C6F7, -F3C4, -F8E8, -F8F9, -E11H7, and S100A12-C5D10, -D10E2, -D10F10, -F5C6 were IgG1 κappa- (κ)-type antibody, and mAbs S100A9-A97A, -A912D, -A912E, -G67B, -C92H, -18H belonged to IgG2b type and processed κ light chains (Table 2). The part sequencing results of S100A8-F8F9, S100A9-C92H, and S100A12-F5C6 mAbs are shown in Figure S3, and the complementarity determining region (CDR) 1, CDR2, and CDR3 are highlighted.

### 2.4. Application of mAbs to Test S100A8, S100A9, and S100A12 Content in the Serum Samples of Piglets

**Figure 4:**
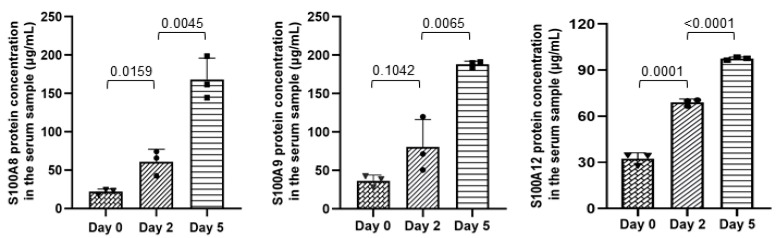
Determination of S100A8, S100A9, and S100A12 proteins in the serum samples of ETEC F4ac-challenged piglets (μg/mL). The serum samples were collected from three piglets on the day before the F4ac challenge (day 0), day 2, and day 5 after the F4ac challenge. Statistical analyses were performed using Student’s *t*-test.

As shown in Figure 4, mAbs S100A8-F8F9, S100A9-C92H, and S100A12-F5C6 have been successfully applied for serum sample detection in piglets, and the concentrations of S100A8, S100A9, and S100A12 proteins were significantly increased in the serum samples of piglets challenged with ETEC F4ac compared to healthy piglets without bacterial challenge. The change in protein content is positively correlated with the severity of symptoms caused by bacterial infection.

## 3. Discussion

S100A8, S100A9, and S100A12 proteins are important members of the S100 protein family and are expressed in multiple cell types in vertebrates. They participate in the regulation of the immune response and can be utilized as robust biomarkers for tracking the process of pathogenic infection or other diseases, such as inflammation, Alzheimer’s disease, RA, IBD, and multiple types of cancers [6,21,22,23,24]. Under certain conditions, they can exert more potent biological functions in the form of dimers (hetero- and homo-), tetramers, or hexamers [11,25,26]. Calprotectin, the stable heterodimer formed by S100A8 and S100A9 monomers, is abundantly expressed in neutrophils and used as a non-invasive diagnostic tool to distinguish gastrointestinal inflammation; it can also attenuate ROS damage and promote autophagy and apoptosis of macrophages, lymphocytes, endothelial cells, and tumor cells [27]. Our previous study proved that the calprotectin content in feces is positively correlated with the degree of inflammation in mice and is useful for evaluating the therapeutic effect of polypyrrole (PPy)-based photothermal nano-antibiotics [28]. S100A12 also exists in a dimer or hexamer form, and Ca^2+^-bound forms of S100A12 are reported to bind directly to soluble tumor necrosis factor (TNF) and affect the efficacy of anti-TNF therapy in patients with juvenile idiopathic arthritis and Crohn’s disease [29]. It also can be used as a target to develop chemotherapeutic drugs or targeted therapeutic molecules, depending on its high affinity with CD36, and show a potential treatment for cancer through the S100A12–CD36 axis [30,31]. In addition to this, the bacteriostatic effects of S100A8, S100A9, and S100A12 proteins in different bacteria, including *Escherichia coli* (*E. coli*), *Helicobacter pylori*, *Clostridium difficile*, *Staphylococcus aureus*, *Klebsiella Pneumoniae*, *Salmonella typhimurium*, etc., have been proposed and verified successively [10,32,33,34,35].

At present, S100A8, S100A9, and S100A12 proteins generated from human or mouse sources have been extensively studied and proven to be powerful in the prevention and treatment of infectious diseases. However, the properties and functions of the porcine S100A8, S100A9, and S100A12 proteins have not been well studied. The genes of S100A8, S100A9 and S100A12 are located in the chromosomal region *4q21*–*q23* comprising three exons and two introns, and are closely linked to *SW512* [2,14,15]. Under normal circumstances, porcine S100A8, S100A9, and S100A12 proteins are preferentially expressed in the bone marrow, spleen, and lymph node, and the mRNA expression levels of these proteins are significantly up-regulated in the serum and tissues of diseased pigs; for example, an increase in S100A8, S100A9, and S100A12 mRNA expression was found in the spleen of piglets infected with *Haemophilus parasuis* [14,15]. In addition, the mRNA expression level of S100A12 is also increased in porcine circovirus-infected PK-15 cells, and it is time-dependent [14]. Therefore, these proteins have the potential to be reliable biomarkers to establish timely detection and diagnostic criteria for the diagnosis of swine disease and the monitoring of anti-inflammatory treatment.

In this study, we expressed and purified the porcine S100A8, S100A9, and S100A12 proteins and confirmed the biological activity of these proteins by their dose-dependent bacteriostatic effects on the F4ac ETEC strain in a certain range. After that, the mAbs against pig-derived proteins S100A8, S100A9 and S100A12 were obtained, and the binding affinities, antigenic specificity, antibody titers, and isotypes of these mAbs were identified. Tests on the serum samples of piglets showed that the above mAbs specifically identified the proteins S100A8, S100A9, and S100A12 in the serum and were able to measure the content of the above proteins in the serum of piglets challenged with bacteria (ETEC F4ac). We also tested the serum samples collected from piglets challenged by viruses (classical swine fever virus and foot-and-mouth disease virus) and achieved similar results. These results provide basic data for evaluating the health status of piglets and establishing a foundation and platform for the subsequent application of S100A8, S100A9, and S100A12 mAbs in swine disease surveillance, further studying the function of porcine S100A8, S100A9, and S100A12 proteins, and developing genetically engineered antibody drugs for preventing or treating gastrointestinal tract or inflammatory diseases in swine.

## 4. Materials and Methods

### 4.1. Animals, Cell Lines, and Culture Conditions

Female BALB/c mice (6–8 weeks of age) were obtained from the Institute of Comparative Medicine of Yangzhou University. Mouse myeloma SP2/0 cells were grown in Dulbecco’s Modified Eagle medium (DMEM, Gibco, Waltham, MA, USA) supplemented with 6% fetal bovine serum (FBS, Gibco, Waltham, MA, USA) and incubated at 37 °C in a 6% CO_2_ humidified atmosphere. Porcine neonatal jejunal IPEC-J2 cells were grown in DMEM supplemented with 10% FBS.

### 4.2. S100A8, S100A9, and S100A12 Gene Cloning and Expression

Total RNA was extracted from the spleen samples of piglets using TRIzol reagent (Vazyme, Nanjing, China) [36]. The *S100A8*, *S100A9*, and *S100A12* genes were amplified using the resulting cDNA and the specific primers listed in Table 3. The PCR products were cloned into pET28a(+) at the *Nhe* I and *Hind* III sites, and the recombinant plasmids pET28a(+)-S100A8, -S100A9, and -S100A12 were identified via restriction enzyme digestion and sequencing. After being transformed into BL21(DE3) *E. coli*, the strains pET28a(+)-S100A8-BL21(DE3), pET28a(+)-S100A9-BL21(DE3), and pET28a(+)-S100A12-BL21(DE3) were constructed and used for recombinant protein expression. After 0.3 mM IPTG (Sigma-Aldrich, St. Louis, MO, USA) induction, the recombinant protein was purified using Protino Ni^+^-TED 2000 Packed Column (MACHEREY-NAGEL, Düren, Germany) and used to immunize six-week-old BALB/c female mice. The purity of the protein was measured by using SDS-PAGE with Coomassie-blue stain. After that, the purified S100A8, S100A9, and S100A12 concentrations were determined through the use of the BCA assay, and S100A8, S100A9, and S100A12 proteins with concentrations of 25, 50, 100, 200, and 300 μg/mL were selected and co-cultured with F4ac (5 × 10^7^ CFUs) for 48 h at 37 °C to explore the in vitro bacteriostatic activities of these proteins. The protein-free treatment group was used as a control. Co-cultures were serially diluted and plated on LB agar to count the number of bacteria. The experiment was repeated three times.

### 4.3. Animals Immunization and Hybridoma Technology to Produce mAbs

In this experiment, 50 μg of S100A8, S100A9, or S100A12 proteins were mixed with complete Freund’s adjuvant (Sigma-Aldrich, St. Louis, MO, USA) for initial immunization and incomplete Freund’s adjuvant (Sigma-Aldrich, St. Louis, MO, USA) for booster immunizations later. All liquids mentioned above were mixed in equal volumes and then subcutaneously injected into female BALB/c mice at two-week intervals. The serum antibody titers against S100A8, S100A9, and S100A12 were detected via indirect enzyme-linked immunosorbent assay (ELISA) using a microtiter plate coated with 6 μg/mL target protein after the third immunization, respectively. Mouse sera (10 μL) collected at each time point were diluted with 490 μL of PBS and then added to the wells of the coated ELISA plate. The OD450 values of each well were measured by using a microplate reader (BioTek BOX 998, Winooski, VT, USA). After that, the selected mice with the highest antibody titer are intraperitoneally injected with 100 μg of S100A8, S100A9, or S100A12 protein for a final antigen boost before cell fusion.

Three days after the final booster immunization, the harvested spleen cell suspension was mixed with mouse myeloma SP2/0 cells at a ratio of 8:1, and polyethylene glycol 1500 (PEG 1500, Roche Diagnostics GmbH, Mannheim, Germany) was used to mediate cell fusion. The fusion cells were cultured in a 96-well plate inoculated with peritoneal macrophages and then selected in HAT (liquid mixture of sodium hypoxanthine, aminopterin, and thymidine, Gibco, Grand Island, NY, USA) medium for ten days, followed by HT (liquid mixture of sodium hypoxanthine and thymidine, Gibco, Paisley, Scotland, UK) medium. The mAbs in the hybridoma supernatant were analyzed via indirect ELISA, and four consecutive positive selection rounds were conducted, selecting the hybridoma showing increased serology response against the S100A8, S100A9, or S100A12 proteins through the use of a limited dilution assay. The positive hybridoma cells are harvested and intraperitoneally injected into pristane-treated mice to produce mAbs [36,37].

### 4.4. Construction of Stable Cell Lines for S100A8, S100A9, and S100A12 Protein Expression

The *S100A8*, *S100A9*, and *S100A12* genes were amplified and then cloned into the pcDNA3.1(+) vector at the *Nhe* I and *Hind* III sites. The resultant plasmids were transfected into IPEC-J2 cells using Lipofectamine 2000 Reagent (Thermo Fisher Scientific, Waltham, MA, USA). 500 μg/mL Geneticin™ Selective Antibiotic (G418 Sulfate, Thermo Fisher Scientific, Waltham, MA, USA) was used to select for cells that stably expressed S100A8, S100A9, and S100A12 proteins.

### 4.5. Characterization of mAbs against the S100A8, S100A9, and S100A12 Proteins

The mAbs were harvested from the supernatant of ascites and purified by using Protein A Agarose (Fast Flow, Beyotime, Shanghai, China). The production of S100A8, S100A9, and S100A12-specific antibodies was confirmed via IFA using pcDNA3.1(+)-S100A8-IPEC-J2, pcDNA3.1(+)-S100A9-IPEC-J2 and pcDNA3.1(+)-S100A12-IPEC-J2 cells, respectively. The cells were harvested and deposited onto a glass slide (NEST, Shanghai, China) overnight at 37 °C. Cells of 60–70% confluency were fixed in 4% formaldehyde at room temperature for 8–10 min, the slides were washed three times with PBS + 0.1% Triton X-100 (PBST), and blocked in PBST containing 5% BSA for 1 h at 37 °C. After three washes, the slides were incubated with mAbs (S100A8-F8F9 mAb (1:1000), S100A9-C92H mAb (1:1000), S100A12-F5C6 mAb (1:500)) for 1 h at 37 °C, and then washed the slides in PBST, followed by incubating with 488 or 549-Goat Anti-Mouse IgG for 30 min at room temperature. The slides were stained with DAPI (cat# D1306, Invitrogen, Waltham, MA, USA) for 10–15 min, washed, and mounted with coverslips before imaging. DyLight 488-Goat Anti-Mouse IgG (1:200, Abbkine, Wuhan, China) was used for S100A8 detection, and images were captured using a Leica TCS SP8 STED confocal microscope (Wetzlar, Germany). Dylight 549-Goat Anti-Mouse IgG (1:200, Abbkine, Wuhan, China) was used for S100A9 and S100A12 detection, and images were captured using a Fluorescent Inverted microscope (IX51; Olympus, Tokyo, Japan).

SDS-PAGE and Western blotting were used to assess the purity and specificity of the mAbs. In this experiment, 1 μg of the protein sample was loaded into each well of the SurePAGE™ precast gel (4–20%, GenScript, Nanjing, China), and then the protein was transferred onto a PVDF membrane for Western blot analysis. The blot was incubated with S100A8-F8F9 mAb (1:1000 dilution), S100A9-C92H mAb (1:1000 dilution), or S100A12-F5C6 mAb (1:1000 dilution), respectively. The binding affinities of these mAbs were determined using the ELISA assay, as described previously [38]. The absorption of samples containing S100A8-F8F9, S100A9-C92H, or S100A12-F5C6 mAbs, and the target protein (S100A8, S100A9, or S100A12) at various extents, were measured at the wavelength of 450 nm. A four-parameter logistic curve fit was used to plot the binding curves. The purified antibody titers were tested via indirect ELISA, as mentioned before, and the antibody protein concentrations were measured using the bicinchoninic acid (BCA, Beyotime, Shanghai, China) assay. The immunoglobulin subtype of the collected antibodies was determined through the SBA Clonotyping System-HRP (Southern-Biotech, Birmingham, AL, USA) according to the manufacturer’s instructions [39]. In addition, the total RNA from the above-mentioned hybridoma cells was extracted and used to synthesize cDNA and amplify variable regions of mAbs using nested PCR, and the sequences of mAbs were analyzed using the IMGT mouse genome analysis tool (http://www.imgt.org/about/immuno-informatics.php, accessed on 13 January, 2024) after sequencing.

### 4.6. Determination of S100A8, S100A9, and S100A12 Protein Contents in the Serum Using mAbs

The protein content of S100A8 in the serum samples of piglets was quantified by using the double-antibody sandwich ELISA using plates coated with mAbs against S100A8. The protein concentration of mAbs was determined through the use of a prior BCA assay. The serum samples were stored in our lab and collected from the piglets challenged with ETEC F4ac or not [40]. The whole blood was stored immediately after collection at 4 °C for six to eight hours, and centrifuging was conducted at 1000–2000× *g* for 20 min in a refrigerated centrifuge. The resulting supernatant was the test serum sample. After incubating with the test serum samples, the plates were rewashed, followed by the addition of HRP-Goat anti-Porcine IgG (H+L, Biodragon, Suzhou, China). The follow-up operation was carried out according to the ELISA method [41], and a standard curve was generated from serially diluted S100A8 protein with known concentrations. The OD_450_ value of the sample was assigned to the standard curve and used to calculate the content of S100A8 protein in the serum. The same procedure applies to the detection of S100A9 and S100A12 proteins in the serum as well.

## Figures and Tables

**Table 1:** The ELISA titers of mAbs against S100A8, S100A9, and S100A12 proteins.

Cells	Titers of Cell Culture Supernatants	Titers of Ascites
S100A8-C6D2	0.4 × 10^3^	0.4 × 10^5^
S100A8-C6F6	0.4 × 10^3^	6.4 × 10^4^
S100A8-C6F7	1.6 × 10^3^	0.8× 10^5^
S100A8-F3C4	0.8 × 10^3^	0.4 × 10^5^
S100A8-F8E8	1.6 × 10^3^	0.8 × 10^5^
S100A8-F8F9	1.6 × 10^3^	0.8 × 10^5^
S100A8-E11H7	0.8 × 10^3^	1.6 × 10^5^
S100A9-A97A	0.8 × 10^3^	1.6 × 10^5^
S100A9-A912E	0.8 × 10^3^	1.6 × 10^5^
S100A9-A912D	0.8 × 10^3^	0.8 × 10^5^
S100A9-G67B	0.4 × 10^3^	0.8 × 10^5^
S100A9-C92H	0.8 × 10^3^	3.2 × 10^5^
S100A9-18H	0.8 × 10^3^	1.28 × 10^5^
S100A12-C5D10	0.2 × 10^3^	0.5 × 10^4^
S100A12-D10E2	0.4 × 10^3^	0.8 × 10^4^
S100A12-D10F10	0.2 × 10^3^	0.4 × 10^4^
S100A12-F5C6	0.2 × 10^3^	2.56 × 10^4^

**Table 2:** Isotypes of hybridoma-derived mAbs against S100A8, S100A9, and S100A12 proteins *.

	Ig	IgA	IgM	IgG1	IgG2a	IgG2b	IgG3	Kappa	Lambda
S100A8-C6D2	0.889	0.131	0.089	3.095	0.104	0.086	0.087	0.384	0.079
S100A8-C6F6	0.917	0.096	0.105	3.1	0.163	0.109	0.084	0.369	0.086
S100A8-C6F7	0.929	0.126	0.166	3.157	0.158	0.09	0.087	0.381	0.082
S100A8-F3C4	1.096	0.095	0.117	3.071	0.15	0.138	0.084	0.551	0.115
S100A8-F8E8	0.928	0.093	0.09	3.003	0.116	0.35	0.104	0.368	0.096
S100A8-F8F9	1.19	0.085	0.106	3.136	0.116	0.177	0.101	0.642	0.076
S100A8-E11H7	1.385	0.081	0.096	3.157	0.083	0.152	0.12	0.746	0.071
S100A9-A97A	1.495	0.081	0.1	0.091	0.09	2.764	0.089	0.961	0.128
S100A9-A912E	1.614	0.089	0.102	0.091	0.09	2.845	0.09	0.999	0.091
S100A9-A912D	1.439	0.113	0.107	0.107	0.109	2.728	0.101	0.913	0.092
S100A9-G67B	1.671	0.089	0.088	0.116	0.1	2.832	0.101	0.93	0.085
S100A9-C92H	1.687	0.132	0.097	0.11	0.101	2.893	0.122	1.168	0.082
S100A9-18H	1.531	0.082	0.097	0.094	0.088	2.826	0.093	0.943	0.09
S100A12-C5D10	0.481	0.08	0.097	2.859	0.088	0.127	0.092	0.293	0.095
S100A12-D10E2	0.505	0.125	0.096	2.927	0.095	0.09	0.086	0.31	0.093
S100A12-D10F10	0.514	0.089	0.124	2.898	0.117	0.095	0.092	0.291	0.081
S100A12-F5C6	0.588	0.091	0.098	2.903	0.087	0.096	0.101	0.346	0.082

* The type of mAbs were red-labelled.

**Table 3:** Primers used in this study.

Primers	Primer Sequence ^1^	Length (bp)
*S100A8*-F	5′-CTAGCTAGCGCCACCATGCTGACGG-3′	270 bp
*S100A8*-R	5′-CCGAAGCTTCTCTTTGTGGATG-3′	
*S100A9*-F	5′-CCGCTAGCGCCACCATGGCGGACC-3′	435 bp
*S100A9*-R	5′-CGCAAGCTTGTGGCTGTGGCCA-3′	
*S100A12*-F	5′-CTAGCTAGCGCCACCATGACTAAGC-3′	279 bp
*S100A12*-R	5′-CCAAGCTTCTCCTTGTGGATGTTG-3′	

^1^ Restriction sites are underlined.

## Data Availability

The source data are provided within this article, and all reagents generated in this study are available from the corresponding author upon reasonable request.

## Supplementary Materials

The following supporting information can be downloaded at: https://www.mdpi.com/article/10.3390/ijms25021029/s1.
