# Supplementary material for: Generation and Application of Monoclonal Antibodies against Porcine S100A8, S100A9, and S100A12 Proteins Using Hybridoma Technology
Source: Int J Mol Sci. 2024 Jan 14;25(2):1029. doi: 10.3390/ijms25021029 (PMC10816078; doi:10.3390/ijms25021029)
Supplement: Supplementary file 1 [file ijms-25-01029-s001.zip › ijms-2773204-supplementary.pdf]

Supplementary Materials

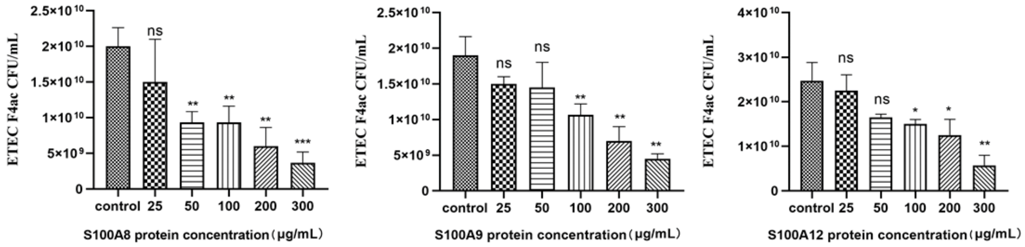

Figure S1. Bacterial load statistics of different concentrations of S100A8, S100A9, and S100A12 proteins (25, 50, 100, 200, and 300 μg/mL) co-cultured with F4ac for 48 h (the relative value between different groups and the control (without protein treatment) were analyzed by SPSS 16.0 software (SPSS Inc., USA) using student's t-test, and ns represents no statistical difference between the data, * represents $P<0.05$, ** represents $P<0.01$, *** represents $P<0.001$ and there are significant statistical differences between the data). All proteins expressed by the prokaryotic expression system in this experiment can perform an antimicrobial function *in vitro* and the bacteriostatic effects on the F4ac ETEC strain were dose-dependent in a certain range.

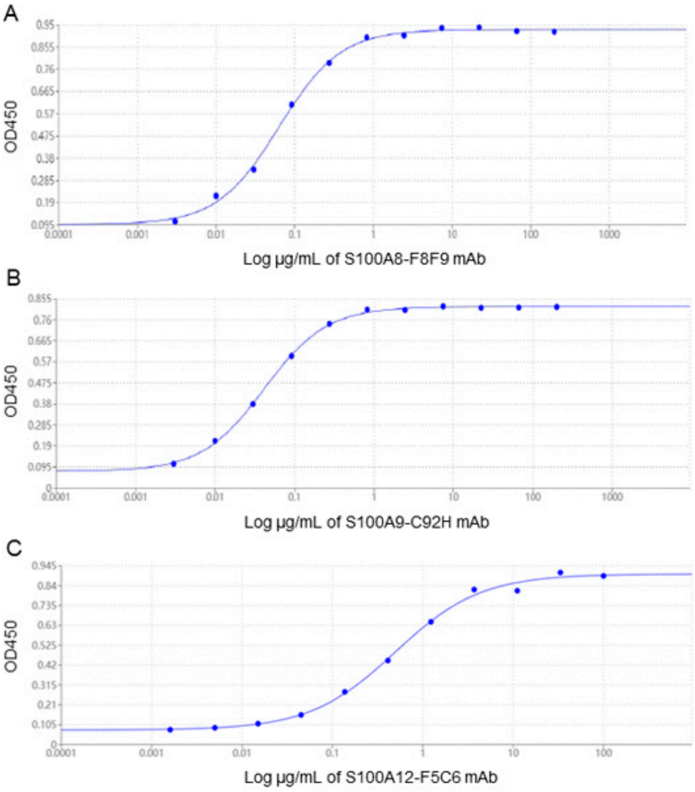

Figure S2. Determination of S100A8-F8F9, S100A9-C92H, and S100A12-F5C6 mAbs antibody relative binding affinities using the ELISA assay. The binding curve was plotted using a four-parameter logistic curve fit, the X-axis shows the logarithmic concentration of antibodies, and the y-axis shows the absorbance at 450 nm.

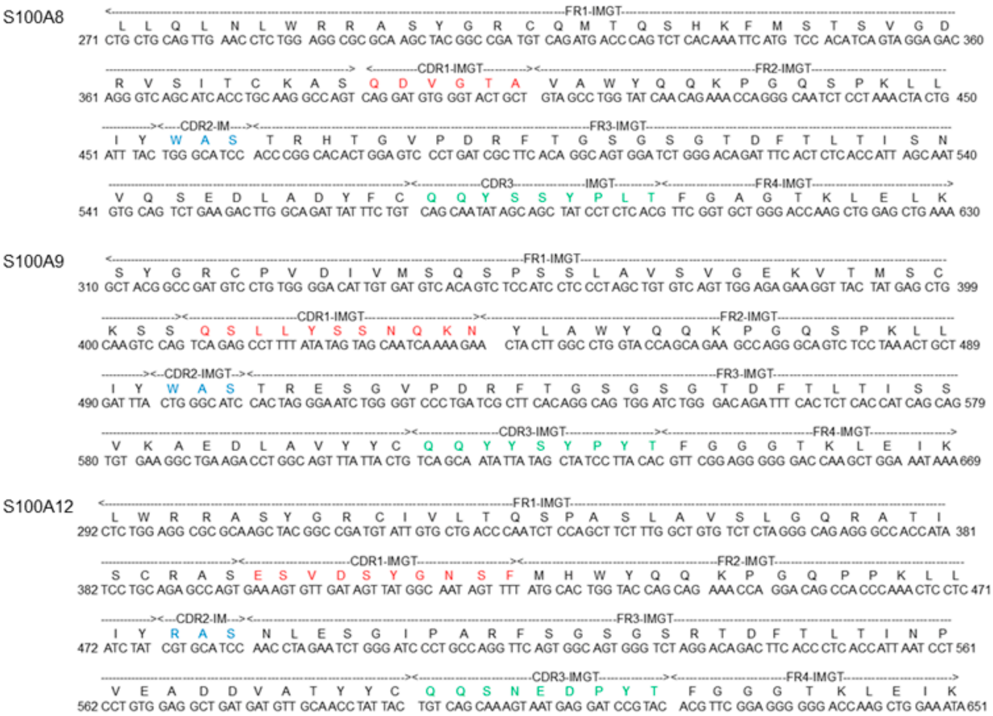

Figure S3. The sequences encoding the framework regions (FR) and CDR of the S100A8-F8F9, S100A9-C92H, and S100A12-F5C6 mAbs. The CDR1, CDR2, and CDR3 regions are highlighted in red, blue, and green, respectively.
